# Association of Antenatal Corticosteroids with Neonatal Outcomes among Very Preterm Infants Born to Mothers with Clinical Chorioamnionitis: A Multicenter Cohort Study

**DOI:** 10.3390/children11060680

**Published:** 2024-06-03

**Authors:** Qingqing Lin, Yanchen Wang, Ying Huang, Wei Zhu, Siyuan Jiang, Xinyue Gu, Jianhua Sun, Shoo K. Lee, Wenhao Zhou, Deyi Zhuang, Yun Cao, on behalf of Chinese Neonatal Network

**Affiliations:** 1 Division of Neonatology, Xiamen Children’s Hospital (Children’s Hospital of Fudan University at Xiamen), Xiamen 361006, China; linqingqing1210@163.com (Q.L.); huangying2912@sina.com (Y.H.); zhuweixm@163.com (W.Z.); 2 NHC Key Laboratory of Neonatal Diseases, Fudan University, Children’s Hospital of Fudan University, Shanghai 201102, China; wang861@mcmaster.ca (Y.W.); jiangsiyuan@fudan.edu.cn (S.J.); xgu5@tulane.edu (X.G.); 3 Department of Health Research Methods, Evidence, and Impact, McMaster University, Hamilton, ON L8S 4L8, Canada; 4 Division of Neonatology, Children’s Hospital of Fudan University, Shanghai 201102, China; 5 Department of Neonatology, Shanghai Children’s Medical Center, School of Medicine, Shanghai Jiao Tong University, Shanghai 200127, China; sunjianhua@scmc.com.cn; 6 Maternal-Infants Care Research Centre, and Department of Pediatrics, Mount Sinai Hospital, Toronto, ON M5G 1X5, Canada; shoo.lee@sinaihealth.ca; 7 Department of Pediatrics, The University of Toronto, Toronto, ON M5T 3M7, Canada; 8 Guangzhou Women and Children’s Medical Center, Guangzhou Medical University, Guangzhou 510623, China; zhouwenhao@fudan.edu.cn; 9 Fujian Key Laboratory of Neonatal Diseases, Xiamen Key Laboratory of Neonatal Diseases, Xiamen Children’s Hospital (Children’s Hospital of Fudan University at Xiamen), Xiamen 361006, China

**Keywords:** antenatal corticosteroids, chorioamnionitis, preterm birth infants, prematurity, mortality

## Abstract

The objective of this study was to assess the relationship of ACS with neonatal outcomes among very preterm infants born to mothers with clinical chorioamnionitis in China. This was a multicenter retrospective cohort study. Study participants included infants born at <32 weeks’ gestation with clinical chorioamnionitis and registered in the Chinese Neonatal Network from 1 January 2019 to 31 December 2020. Infants were divided into two groups: any amount of ACS or no administration of ACS. Multivariable generalized linear models using generalized estimating equations were used to assess the association between ACS and neonatal outcomes among the study population. We identified 2193 infants eligible for this study; 1966 (89.6%) infants had received ACS therapy, and 227 (10.4%) had not received any ACS therapy. Among very preterm infants born to mothers with clinical chorioamnionitis, any ACS usage was significantly associated with decreased risks of early death (aRR 0.56, 95% CI 0.32, 0.99) and severe ROP (aRR 0.51, 95% CI 0.28, 0.93) after adjustment for maternal hypertension, gestational age at birth, Caesarean section, being inborn, and administration of systemic antibiotics to the mother within 24 h before birth. In addition, out of the 2193 infants, the placentas of 1931 infants underwent pathological examination with recorded results. Subsequently, 1490 of these cases (77.2%) were diagnosed with histological chorioamnionitis. In 1490 cases of histologic chorioamnionitis, any ACS usage was significantly related to decreased risks of overall mortality (aRR 0.52, 95% CI 0.31, 0.87), severe ROP (aRR 0.47, 95% CI 0.25, 0.97), and respiratory distress syndrome (aRR 0.52, 95% CI 0.31, 0.87). We concluded that any ACS was associated with reduced risks for neonatal early death and severe ROP among very preterm infants born to mothers with clinical chorioamnionitis.

## 1. Introduction

Chorioamnionitis is a common cause of preterm birth [1,2]. Both clinical and histologic chorioamnionitis have been suggested as risk factors for adverse neonatal outcomes [3,4].

Antenatal corticosteroids (ACS) have been proven to greatly improve outcomes for preterm infants, including reduction of neonatal death, respiratory distress syndrome (RDS), and intraventricular hemorrhage (IVH) [5]. ACS has been strongly recommended for routine administration to women within seven days being at risk of preterm delivery from 24^+0^ to 34^+6^ weeks of gestation [6,7,8,9,10]. However, there is still some conflicting evidence related to the administration of ACS in specific conditions, particularly in mothers with chorioamnionitis. Recent systematic reviews revealed significantly lower risks of neonatal mortality and morbidities in the ACS group compared to the non-ACS group among infants with maternal clinical or histologic chorioamnionitis [2,11,12]. However, the WHO Recommendations on Interventions to Improve Preterm Birth Outcomes stated that they do not recommend ACS therapy in women with chorioamnionitis who will likely deliver preterm [10] because of possible increased risks of maternal infectious morbidity and neonatal mortality in developing countries [13]. The WHO Recommendation was a conditional recommendation with low evidence. Furthermore, guidelines in developed countries did not report recommendations on ACS usage among mothers with chorioamnionitis [6,7].

Our primary objective in this study was to examine the association of ACS with neonatal outcomes among very preterm infants born to mothers with chorioamnionitis in China.

## 2. Materials and Methods

### 2.1. Study Design and Settings

This was a retrospective cohort study, using the Chinese Neonatal Network (CHNN) database [14]. The CHNN was established in 2018 and maintains a standardized clinical database of all preterm infants born at <32 weeks’ gestation admitted to 70 NICUs to monitor outcomes and care practices. Data collection and transmission from each unit were approved by the local research ethics board. This study was approved by the ethics review board of the Children’s Hospital of Fudan University (No. 2018-296), and the ethical approval date was 19 December 2018. Informed consent was waived, as only de-identified patient data were accessed.

### 2.2. Study Population

Infants were included in our study if they were (1) born at <32 weeks’ gestation with clinical chorioamnionitis and (2) admitted to CHNN NICUs between 1 January 2019 and 31 December 2020. Infants were excluded if they (1) had major congenital malformations, (2) were missing information on antenatal corticosteroids, or (3) were discharged against medical advice (DAMA). Major congenital malformations were defined as life-threatening congenital malformations leading to death or severe neurodevelopmental impairments [15]. DAMA was defined as the termination of therapy in the NICU by parents or caregivers before the physician suggested discharge [16]. Readmissions and transfers between CHNN-participating hospitals were tracked. However, stillbirths, delivery-room deaths, and infants transferred to non-participating hospitals within 24 h after birth were not captured by the database.

### 2.3. Definitions of Chorioamnionitis and Gestational Age

Chorioamnionitis included both clinical chorioamnionitis and histologic chorioamnionitis. The diagnostic criteria of clinical chorioamnionitis included maternal temperature greater than 38.0 °C before labor in the absence of another focus for infection with two or more of the following: uterine tenderness, malodorous vaginal discharge, maternal leukocytosis (white-blood-cell count 15,000 cells/L), maternal tachycardia (100 beats/min), and fetal tachycardia (160 beats/min) [2]. In addition, histologic chorioamnionitis was defined as the presence of acute inflammatory change of polymorphonuclear leukocyte infiltration in any part of the amnion, chorionic decidua, umbilical cord, or chorionic plate based on a pathologic review of the placenta by pathologists at each participating facility [17]. 

Gestational age at birth (GA) was determined using the hierarchy of best obstetric estimates based on prenatal ultrasound, menstrual history, obstetric examination, or all three. The term “small for gestational age” (SGA) was defined as birthweight < 10th percentile for the gestational age at birth and sex according to the Chinese neonatal-birthweight values [18]. The term “inborn” referred to infants born at a level-III maternity hospital.

### 2.4. Data Collection

The data-collection process was described in previous publications from CHNN [14,19,20]. The CHNN coordinating center was responsible for data quality control, and data were regularly checked, corrected, and audited to ensure high data quality [19,20]. 

### 2.5. Exposures

ACS exposure was defined as maternal receipt of at least one dose of dexamethasone or betamethasone before delivery. According to maternal administration of ACS before delivery, infants were divided into two groups: any amount of ACS (exposed group) and no administration of ACS (unexposed group). In subgroup analysis, the ACS groups were divided into three groups: a single partial course, a single complete course, and repeated courses. A single complete course of antenatal corticosteroids was defined as four doses of 6 mg dexamethasone at a 12-h interval before birth or two doses of 12 mg betamethasone at a 24-h interval. A single partial course was defined as one to three doses of dexamethasone or one dose of betamethasone. Repeated ACS usage was defined as any number of steroids given in addition to one complete course of ACS.

### 2.6. Outcomes

Mortality was defined as the death of an infant before discharge. Early death was defined as neonatal death within seven days after birth. Necrotizing enterocolitis (NEC) was diagnosed according to Bell’s criteria (stage 2 or greater) [21]. Bronchopulmonary dysplasia (BPD) was defined as mechanical ventilation or oxygen dependency at 36 weeks’ postmenstrual age or discharge [22]. Brain injury included severe IVH or cystic periventricular leukomalacia (cPVL). Severe IVH was defined as IVH grade III or above according to Papile’s criteria [23]. cPVL was defined as the presence of periventricular cysts on cranial ultrasound or MRI scans before discharge. Severe retinopathy of prematurity (ROP) was defined as ROP stage III or above according to the International Classification of ROP [24]. Sepsis was defined as positive blood or cerebrospinal fluid culture with antibiotic treatment lasting ≥5 days. Early sepsis was defined as culture-proven sepsis within 72 h after birth. RDS was diagnosed based on clinical presentation and characteristic radiographic appearance.

### 2.7. Statistical Analyses

Maternal and infant characteristics were summarized descriptively and compared between each ACS group and the non-ACS group. To determine the association of ACS with neonatal outcomes among infants born to mothers with clinical chorioamnionitis, multivariable Poisson models estimated by generalized estimating approaches (GEE) were employed to account for the cluster effect within CHNN-participating hospitals. These models were adjusted for maternal and infant characteristics, including maternal hypertension, gestational age at birth, Caesarean section, inborn status, and the administration of systemic antibiotics to mothers within 24 h before birth. Multivariable Poisson regressions with the same covariates were performed among groups given different courses of ACS. Sensitivity analyses were performed among infants born to mothers with histologic chorioamnionitis to assess the robustness of the result. 

We applied SAS Version 9.4 (SAS Institute, Cary, NC, USA) to perform all data management and analyses. The statistical-significance level was set at *p* < 0.05 with the two-tailed test.

## 3. Results

**Figure 1:**
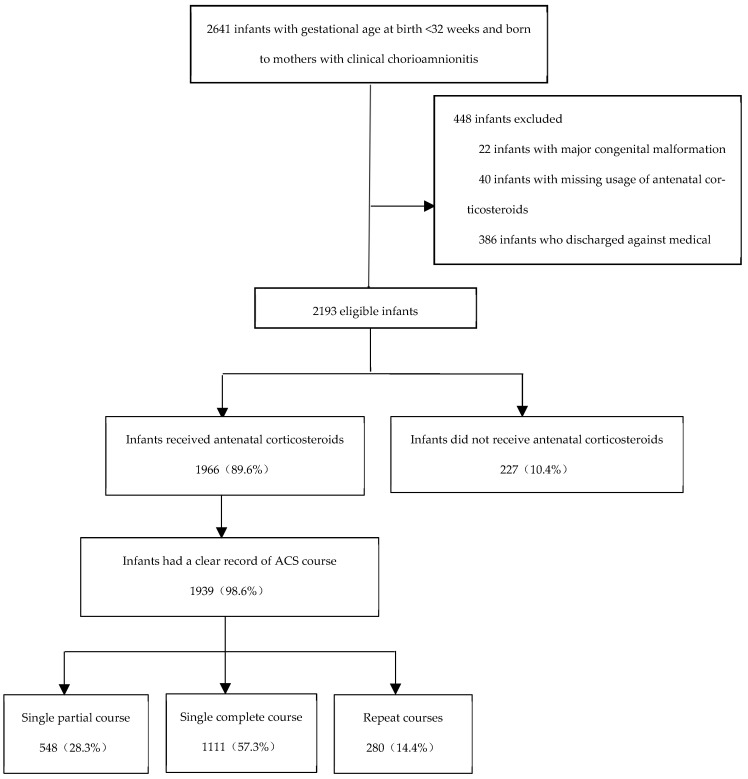
Study Population.

Initially, 2641 infants born to mothers diagnosed with clinical chorioamnionitis and included in the Chinese Neonatal Network database were considered for eligibility in this study (Figure 1). After 448 infants were excluded, the remaining 2193 infants constituted the study population. Among them, 1966 (89.6%) infants had received some amount of ACS therapy (Figure 1). There was no significant difference in the level of ACS usage across different gestational ages at birth (Table S1). As shown in Table 1, of the 2193 infants diagnosed with clinical chorioamnionitis, the placentas of 1931 infants underwent pathological examination with recorded results. Among them, 1490 cases (77.2%) were found to have histological chorioamnionitis.

### 3.1. Association of ACS with Neonatal Outcomes among Infants Born to Mothers with Clinical Chorioamnionitis

Infant and maternal characteristics associated with clinical chorioamnionitis are shown in Table 1. The median GA of these 2193 infants was 30 weeks, and the mean birth weight was 1343 g (Table 1). Infants exposed to any amount of ACS had significantly higher gestational ages at birth and were more likely to be inborn compared to infants not administered ACS (Table 1). Mothers with clinical chorioamnionitis who had been given ACS were more likely to be given systemic antibiotics within 24 h before birth (Table 1).

Compared to infants unexposed to ACS, infants exposed to any ACS also showed lower incidences of mortality, early death, RDS, brain injury, BPD, and severe ROP (Table 2). Table 3 shows the results of multivariable Poisson regressions using the GEE approaches. Compared to the non-ACS group, the any ACS group showed decreased risks of severe ROP (aRR 0.51, 95% CI 0.28, 0.93) and early death (aRR 0.56, 95% CI 0.32, 0.99).

### 3.2. Association of ACS with Neonatal Outcomes among Infants Born to Mothers with Histologic Chorioamnionitis

Among 1490 infants born to mothers with histologic chorioamnionitis, 1350 (90.6%) infants received ACS therapy, and 140 (9.4%) infants did not receive any ACS therapy (Table S2). Infant and maternal characteristics among the subgroup with histologic chorioamnionitis are shown in Table S2. Compared to those not exposed to ACS, infants exposed to any amount of ACS had significantly higher gestational age at birth, rates of inborn status, and rates of administration of systemic antibiotics to mothers within 24 h before birth (Table S2). 

Table S3 showed that the infants born to mothers with histologic chorioamnionitis had similar results on univariable analyses compared to the overall study population. In multivariable models, ACS was significantly associated with decreased risks of overall mortality (aRR 0.52, 95% CI 0.31, 0.87), severe ROP (aRR 0.47, 95% CI 0.25,0.97), and RDS (aRR 0.52, 95% CI 0.31, 0.87) among infants born to mothers with histologic chorioamnionitis (Table 3).

### 3.3. Association of Different ACS Courses with Neonatal Outcomes among Infants with Clinical Chorioamnionitis

Among 1966 infants exposed to ACS, 1939 infants (98.6%) had complete information on ACS courses. Of these 1939 infants, 548 (28.3%) infants received a single partial course of ACS, 1111 (57.3%) infants received a single complete course of ACS, and 280 (14.4%) infants received repeated courses of ACS (Figure 1). Infants who received a single complete course of ACS showed lower rates of early death, sepsis, brain injury, BPD, and NEC than those who received a single partial course or repeated courses of ACS (Table S4). As shown in Table 4, infants who received a single complete course of ACS showed lower risks of BPD (aRR 0.83, 95% CI 0.71, 0.98) and early death (aRR 0.46, 95% CI 0.25, 0.85) compared to those who did not receive ACS.

## 4. Discussion

Our study is one of the largest cohort studies from China to examine the association of ACS therapy with neonatal outcomes of very preterm infants born to mothers with clinical chorioamnionitis. We found a high rate (89.6%) of ACS administration to women with clinical chorioamnionitis at risk for preterm birth in China. The decision against administering antenatal corticosteroids to mothers with clinical chorioamnionitis may be frequently influenced by the lack of adequate time for administration before delivery. After adjustment for potential confounders, ACS therapy was associated with reduced risks for early death and severe ROP among very preterm infants born to mothers with clinical chorioamnionitis. Furthermore, among infants born to mothers with histological chorioamnionitis, ACS therapy reduced the risks of mortality, severe ROP, and RDS. Compared to infants whose mothers did not receive ACS, a single complete course of ACS reduced the risks of early death and BPD. 

Our results were consistent with the findings of a meta-analysis of nine observational studies in high-income countries showing that ACS was associated with reduced mortality (OR 0.45, 95% CI 0.36–0.56) among preterm infants with maternal histologic chorioamnionitis, but not clinical chorioamnionitis (OR 0.77, 95% CI 0.36–1.65) [2]. Our research also showed that in clinical chorioamnionitis, ACS was associated with a reduced risk of early death, which had not been previously reported in the literature [2]. Several mechanisms may explain the advantages of the administration of ACS therapy to mothers with chorioamnionitis, including acceleration of fetal lung maturation and fetal organ maturation [5,6], more stable hemodynamics, cerebral capillary stabilization, and organotoxic cytokine suppression [25]. As a result, ACS therapy is associated with a reduced risk of neonatal early death among infants born to mothers with clinical chorioamnionitis. Even if the condition is finally determined to be histologic chorioamnionitis, ACS is beneficial to infants, resulting in reduced mortality.

Our study found that ACS was associated with a decreased incidence of severe ROP in NICU infants born to mothers with both clinical and histological chorioamnionitis. Recently, a meta-analysis showed the association between ACS exposure and the development of ROP in preterm infants, including 63 cohort studies involving infants born at gestational ages from 25 to 32 weeks [26]. The results showed ACS exposure was associated with significantly lower odds of ROP progression (aOR 0.48, 95% CI 0.26–0.89). ACS has long been suggested to modulate fetal maturity and thus may accelerate the maturation of the retinal vasculature as well [27]. In addition, ACS may reduce the risks of RDS and oxygen therapy, which have long been recognized as risk factors for ROP [28,29]. Furthermore, a systemic review suggested that a prenatal “pre-phase” of ROP sensitized the retina to in-utero inflammatory factors and subsequently triggered dysregulation of angiogenesis [30]. This evidence supported the protective effects of ACS on severe ROP in our study.

Our results showed that a single complete course may reduce the risks of early death and BPD compared to non-administration of ACS. Our results were in line with the results of a previous study that found that optimal administration of ACS was associated with a reduction in the risks of death and severe BPD (aOR 0.03, 95% CI 0.01–0.42) among in-hospital preterm singletons [31]. Due to the anti-inflammatory effect of ACS, optimal usage of ACS can reduce intrauterine inflammation among mothers with chorioamnionitis. Reduction of intrauterine inflammation can decrease dysregulation of growth factors, mesenchymal structural proteins, and signaling pathways, which may slow structural changes in the fetal lung, leading to a reduced risk of BPD [32]. Our findings also concur with the results of previous studies and with guidelines recommending that a complete course is optimal for ACS management within one to seven days before birth, even in cases of clinical chorioamnionitis [5,33,34,35].

This was one of the largest cohort studies in China examining the association of ACS with neonatal outcomes exclusively among infants with exposure to clinical chorioamnionitis. In this study, sensitivity analyses were conducted for histologic chorioamnionitis. However, there were several limitations in our study. First, we did not collect information about the timing of ACS administration relative to the diagnosis of clinical chorioamnionitis. Second, our cohort was a hospital-based cohort, with stillbirths and delivery-room deaths excluded. Third, not all births to mothers with clinical chorioamnionitis involved pathological examination of the placenta, which may lead to an underestimation of the rate of histologic chorioamnionitis. Fourth, 386 infants (14.6%) with DAMA were excluded from this study, which might have introduced selection bias. The rate of ACS usage among infants with DAMA (80.1%) was lower than that among infants with complete care (88%), but the lack of outcome information for DAMA infants made it impossible to assess the association between ACS and outcomes among these infants. Fifth, some unmeasured confounders (such as antenatal bleeding, incidental short cervix, and incidental open cervix) could not be adjusted for in the statistical analysis, which may bias the results [36].

## 5. Conclusions

For very preterm infants born to mothers with clinical chorioamnionitis, ACS was significantly associated with reduced risks of early death and severe ROP. More specifically, a single complete course of ACS was associated with decreased risks of early death and BPD compared to non-administration of ACS.

## Figures and Tables

**Table 1:** Comparisons of maternal and infant characteristics between very preterm infants with or without administration of antenatal corticosteroids born to mothers with clinical chorioamnionitis.

	Total (N = 2193)	Non-ACS Group (N = 227)	ACS Group (N = 1966)	*p*-Value
**Maternal Information**				
Maternal age, mean (std)	31.45 (4.69)	31.44 (4.65)	31.45 (4.69)	0.964
Hypertension, n/N (%)	204/2191 (9.3%)	18/226 (8.0%)	186/1965 (9.5%)	0.462
Diabetes, n/N (%)	482/2190 (22.0%)	44/226 (19.5%)	438/1964 (22.3%)	0.330
Caesarean section, n/N (%)	1195/2193 (54.5%)	112/227 (49.3%)	1083/1966 (55.1%)	0.099
Primigravida, n/N (%)	1209/2181 (55.4%)	125/226 (55.3%)	1084/1955 (55.4%)	0.968
Maternal fever, n/N (%)	346/2074 (16.7%)	40/205 (19.5%)	306/1869 (16.4%)	0.252
Systemic antibiotics given within 24 h before birth, n/N (%)	1654/2147 (77.0%)	135/218 (61.9%)	1519/1929 (78.7%)	<0.001
Histological chorioamnionitis, n/N (%)	1490/1931 (77.2%)	140/191 (73.3%)	1350/1740 (77.6%)	0.180
**Infants’ Information**				
Gestational age at birth, median (IQR)	30 (28,31)	29 (28,31)	30 (28,31)	0.001
<26 wk	62/2193 (2.8%)	14/227 (6.2%)	48/1966 (2.4%)	0.004
26–27 wk	336/2193 (15.3%)	39/227 (17.2%)	297/1966 (15.1%)	
28–29 wk	793/2193 (36.2%)	86/227 (37.9%)	707/1966 (36.0%)	
30–31 wk	1002/2193 (45.7%)	88/227 (38.8%)	914/1966 (46.5%)	
Birthweight, mean (Std)	1343.36 (302.72)	1306.60 (326.10)	1347.60 (299.70)	0.071
Male, n/N (%)	1262/2191 (57.6%)	132/227 (58.1%)	1130/1964 (57.5%)	0.859
Multiple birth, n/N (%)	577/2193 (26.3%)	50/227 (22.0%)	527/1966 (26.8%)	0.122
Small for gestational Age, n/N (%)	65/2191 (3.0%)	5/227 (2.2%)	60/1964 (3.1%)	0.474
Inborn, n/N (%)	1980/2193 (90.3%)	191/227 (84.1%)	1789/1966 (91.0%)	0.001

Abbreviations: ACS, antenatal corticosteroids; IQR, interquartile range; std: standard deviation.

**Table 2:** Neonatal outcomes of very preterm infants exposed or not exposed to antenatal corticosteroids who were born to mothers with clinical chorioamnionitis.

Outcome	Total (N = 2193)	Non-ACS Group (N = 227)	ACS Group (N = 1966)	*p*-Value
Mortality, n/N (%)	104/2193 (4.7%)	25/227 (11.0%)	79/1966 (4.0%)	<0.0001
NEC ≥ Stage II, n/N (%)	83/2193 (3.8%)	7/227 (3.1%)	76/1966 (3.9%)	0.559
BPD, n/N (%)	578/2187 (26.4%)	79/226 (35.0%)	499/1961 (25.4%)	0.002
Brain injury, n/N (%) ^a^	201/2076 (9.7%)	29/203 (14.3%)	172/1873 (9.2%)	0.020
Severe IVH, n/N (%)	122/2076 (5.9%)	17/203 (8.3%)	105/1873 (5.6%)	0.115
cPVL, n/N (%)	102/2076 (4.9%)	17/203 (8.4%)	85/1873 (4.5%)	0.015
Severe ROP, n/N (%) ^b^	55/1825 (3.0%)	12/175 (6.9%)	43/1650 (2.6%)	0.002
Sepsis, n/N (%)	201/2140 (9.4%)	21/216 (9.7%)	180/1924 (9.4%)	0.861
Early sepsis, n/N (%)	52/2193 (2.4%)	5/227 (2.2%)	47/1966 (2.4%)	0.860
Early death, n/N (%)	66/2187 (3.0%)	16/226 (7.1%)	50/1961 (2.5%)	0.0002
RDS, n/N (%)	1437/2187 (65.7%)	162/225 (72.0%)	1275/1962 (65.0%)	0.036
Apgar score < 7 at 5 min, n/N (%)	88/2129 (4.1%)	16/214 (7.5%)	72/1915 (3.8%)	0.010

^a^ Incidence of brain injury was calculated among infants with neuroimaging results. ^b^ Incidence of severe ROP was calculated among infants given eye examinations in the NICU. Abbreviations: ACS, antenatal corticosteroids; BPD, bronchopulmonary dysplasia; IVH, intraventricular hemorrhage; NEC, necrotizing enterocolitis; PVL, periventricular leukomalacia; RDS, respiratory distress syndrome; ROP, retinopathy of prematurity.

**Table 3:** Association of administration of antenatal corticosteroids and neonatal outcomes among very preterm infants born to mothers with chorioamnionitis.

	Clinical Chorioamnionitis ^a,b^ aRR (95% CI)	Histologic Chorioamnionitis ^a,b^ aRR (95% CI)
Mortality	0.69 (0.44, 1.06)	0.52 (0.31, 0.87)
NEC ≥ Stage II	1.37 (0.71, 2.62)	1.57 (0.52, 4.74)
BPD	0.92 (0.78, 1.08)	0.94 (0.77, 1.16)
Brain injury	0.82 (0.53, 1.27)	0.84 (0.56, 1.28)
Severe IVH	1.01 (0.65, 1.56)	0.81 (0.48, 1.37)
cPVL	0.60 (0.30, 1.18)	0.75 (0.42, 1.37)
Severe ROP	0.51 (0.28, 0.93)	0.49 (0.25, 0.97)
Sepsis	0.96 (0.59, 1.54)	1.14 (0.58, 2.21)
Early sepsis	0.94 (0.35, 2.54)	0.52 (0.25, 1.11)
Early death	0.56 (0.32, 0.99)	1.04 (0.96, 1.12)
RDS	0.99 (0.90, 1.09)	0.52 (0.31, 0.87)

^a^ Infants in the non-ACS group served as a reference. Adjusted relative risk was reported. ^b^ Adjusted for maternal hypertension, gestational age at birth, Caesarean section, being inborn and the administration of systemic antibiotics to the mother within 24 h before birth. Abbreviations: ACS, antenatal corticosteroids; BPD, bronchopulmonary dysplasia; CI, confidence interval; IVH, intraventricular hemorrhage; NEC, necrotizing enterocolitis; aRR, adjusted relative risk; PVL, periventricular leukomalacia; RDS, respiratory distress syndrome; ROP, retinopathy of prematurity.

**Table 4:** Association of different courses of antenatal corticosteroids and neonatal outcomes among very preterm infants born to mothers with clinical chorioamnionitis.

	No ACS	Single Complete Course ^a,b^	Single Partial Course ^a,b^	Repeated Courses ^a,b^
Mortality	1.00 (Reference)	0.65 (0.40, 1.04)	0.71 (0.41, 1.25)	0.83 (0.47, 1.49)
NEC ≥ Stage II	1.00 (Reference)	0.97 (0.50, 1.87)	1.91 (0.91, 4.00)	1.67 (0.80, 3.50)
BPD	1.00 (Reference)	0.83 (0.71, 0.98)	1.03 (0.86, 1.23)	1.00 (0.78, 1.27)
Brain injury	1.00 (Reference)	0.78 (0.47, 1.28)	0.87 (0.56, 1.37)	0.85 (0.54, 1.33)
Severe IVH	1.00 (Reference)	0.91 (0.57, 1.46)	1.16 (0.74, 1.81)	0.92 (0.54, 1.56)
cPVL	1.00 (Reference)	0.57 (0.27, 1.20)	0.54 (0.26, 1.10)	0.90 (0.45, 1.80)
Severe ROP	1.00 (Reference)	0.54 (0.27, 1.07)	0.50 (0.24, 1.04)	0.28 (0.11, 0.70)
Sepsis	1.00 (Reference)	0.88 (0.55, 1.39)	1.13 (0.72, 1.77)	1.00 (0.48, 2.05)
Early sepsis	1.00 (Reference)	0.91 (0.34, 2.48)	0.95 (0.32, 2.82)	1.10 (0.32, 3.73)
Early death	1.00 (Reference)	0.46 (0.25, 0.85)	0.70 (0.36, 1.33)	0.79 (0.36, 1.72)
RDS	1.00 (Reference)	0.98 (0.89, 1.09)	0.99 (0.91, 1.08)	1.02 (0.89, 1.15)

^a^ Infants not exposed to ACS served as a reference. Adjusted relative risks were reported. ^b^ Adjusted for maternal hypertension, gestational age at birth, Caesarean section, being inborn and administration of systemic antibiotics to mother within 24 h before birth. Abbreviations: BPD, bronchopulmonary dysplasia; CI, confidence interval; IVH, intraventricular hemorrhage; NEC, necrotizing enterocolitis; RR, relative risk; PVL, periventricular leukomalacia; RDSan, respiratory distress syndrome; ROP, retinopathy of prematurity.

## Data Availability

The data presented in this study are available on request from the corresponding author. The data are not publicly available due to specific ethical and privacy considerations.

## Supplementary Materials

The following supporting information can be downloaded at: https://www.mdpi.com/article/10.3390/children11060680/s1, Table S1: Usage of antenatal corticosteroids among infants born to mothers with clinical chorioamnionitis; Table S2: Basic characteristics of the study population with histological chorioamnionitis; Table S3: Univariable analysis for association between neonatal outcomes and antenatal corticosteroids among infants born to mothers with histological chorioamnionitis; Table S4: Univariable analysis for association between outcomes and different courses of antenatal corticosteroids among infants born to mothers with clinical chorioamnionitis.
