# Supplementary material for: Association of Antenatal Corticosteroids with Neonatal Outcomes among Very Preterm Infants Born to Mothers with Clinical Chorioamnionitis: A Multicenter Cohort Study
Source: Children (Basel). 2024 Jun 3;11(6):680. doi: 10.3390/children11060680 (PMC11202040; doi:10.3390/children11060680)
Supplement: Supplementary file 1 [file children-11-00680-s001.zip › Table S1.pdf]

Table S1. Usage of antenatal corticosteroids among infants born to mothers with clinical chorioamnionitis

Gestational age at birth (weeks)	Total number of infants	Any ACS n(%)	Single partial course n(%)[a]	Single complete course n(%)[a]	Repeated courses n(%)[a]
<24 wk	12	8/12 (66.7%)	4/8 (50.0%)	4/8 (50.0%)	0/8 (0.0%)
24 wk	11	8/11 (72.7%)	4/8 (50.0%)	4/8 (50.0%)	0/8 (0.0%)
25 wk	39	32/39 (82.1%)	6/31 (19.4%)	19/31 (61.3%)	6/31 (19.4%)
26 wk	114	99/114 (86.8%)	25/98 (25.5%)	59/98 (60.2%)	14/98 (14.3%)
27 wk	222	198/222 (89.2%)	49/198 (24.7%)	123/198 (62.1%)	26/198 (13.1%)
28 wk	374	328/374 (87.7%)	95/321 (29.6%)	179/321 (55.8%)	47/321 (14.6%)
29 wk	419	379/419 (90.5%)	103/375 (27.5%)	229/375 (61.1%)	43/375 (11.5%)
30 wk	495	448/495 (90.5%)	127/443 (28.7%)	238/443 (53.7%)	78/443 (17.6%)
31 wk	507	466/507 (91.9%)	135/457 (29.5%)	256/457 (56.0%)	66/457 (14.4%)
Total	2193	1966/2193 (89.6%)	548/1939 (28.3%)	1111/1939 (57.3%)	280/1939 (14.4%)

[a] Calculate within Infants who had a clear record of ACS course.

Abbreviations: ACS, antenatal corticosteroids
