# Supplementary material for: Association of Antenatal Corticosteroids with Neonatal Outcomes among Very Preterm Infants Born to Mothers with Clinical Chorioamnionitis: A Multicenter Cohort Study
Source: Children (Basel). 2024 Jun 3;11(6):680. doi: 10.3390/children11060680 (PMC11202040; doi:10.3390/children11060680)
Supplement: Supplementary file 1 [file children-11-00680-s001.zip › Table S2.pdf]

Table S2. Basic characteristics of the study population with histological chorioamnionitis

Characteristics	Total (N=1490)	Non-ACS group (N=140)	ACS group (N=1350)	p-value[a]
**Maternal Information**				
Maternal Age, Mean (Std)	31.58 (4.60)	31.60 (4.48)	31.58 (4.62)	0.947
Hypertension, n/N(%)	127/1489 (8.5%)	12/140 (8.6%)	115/1349 (8.5%)	0.985
Diabetes, n/N(%)	355/1488 (23.9%)	31/140 (22.1%)	324/1348 (24.0%)	0.617
Caesarean section, n/N(%)	797/1490 (53.5%)	67/140 (47.9%)	730/1350 (54.1%)	0.160
Primigravida, n/N(%)	846/1482 (57.1%)	85/140 (60.7%)	761/1342 (56.7%)	0.362
Maternal fever, n/ N(%)	225/1427 (15.8%)	22/131 (16.8%)	203/1296 (15.7%)	0.732
Systemic antibiotics given within 24h before birth, n/ N(%)	1119/1471 (76.1%)	83/137 (60.6%)	1036/1334 (77.7%)	<0.001
**Infants Information**				
Gestational age at birth, Median (IQR)	30 (28,31)	29 (28,31)	30 (28,31)	0.017
<26 wk	40/1490 (2.7%)	9/140 (6.4%)	31/1350 (2.3%)	0.017
26-27 wk	229/1490 (15.4%)	25/140 (17.9%)	204/1350 (15.1%)	
28-29 wk	561/1490 (37.7%)	53/140 (37.9%)	508/1350 (37.6%)	
30-31 wk	660/1490 (44.3%)	53/140 (37.9%)	607/1350 (45.0%)	
Birth weight, Mean (Std)	1344.96 (298.25)	1308.40 (339.70)	1348.70 (293.50)	0.178
Male, n/N(%)	864/1489 (58.0%)	81/140 (57.9%)	783/1349 (58.0%)	0.966
Multiple birth, n/N(%)	379/1490 (25.4%)	34/140 (24.3%)	345/1350 (25.6%)	0.742
Small for gestational Age, n/N(%)	47/1489 (3.2%)	2/140 (1.4%)	45/1349 (3.3%)	0.310
Inborn, n/N(%)	1437/1490 (96.4%)	129/140 (92.1%)	1308/1350 (96.9%)	0.013

[a]ACS group comparing with the non-ACS group.
Abbreviations: ACS, antenatal corticosteroids; IQR, interquartile range; std, standard deviation.
