# Supplementary material for: Association of Antenatal Corticosteroids with Neonatal Outcomes among Very Preterm Infants Born to Mothers with Clinical Chorioamnionitis: A Multicenter Cohort Study
Source: Children (Basel). 2024 Jun 3;11(6):680. doi: 10.3390/children11060680 (PMC11202040; doi:10.3390/children11060680)
Supplement: Supplementary file 1 [file children-11-00680-s001.zip › Table S3.pdf]

Table S3. Univariable analysis for the association between neonatal outcomes and antenatal corticosteroids among infants born to mothers with histological chorioamnionitis

**Outcomes**	**Total (N=1490)**	**Non-ACS group （N=140）**	**ACS group (N=1350)**	***p*-value**[a]
Mortality, n/N(%)	67/1490 (4.5%)	19/140 (13.6%)	48/1350 (3.6%)	<0.0001
NEC≥Stage II, n/N(%)	57/1490 (3.8%)	4/140 (2.9%)	53/1350 (3.9%)	0.530
BPD, n/N(%)	381/1489 (25.6%)	46/140 (32.9%)	335/1349 (24.8%)	0.038
Brain Injury, n/N(%)[b]	125/1413 (8.8%)	17/122 (13.9%)	108/1291 (8.4%)	0.038
Severe IVH, n/N(%)	75/1413 (5.3%)	13/122 (10.7%)	62/1291 (4.8%)	0.006
cPVL, n/N(%)	66/1413 (4.7%)	8/122 (6.6%)	58/1291 (4.5%)	0.302
Severe ROP, n/N(%)[c]	41/1210 (3.4%)	7/98 (7.1%)	34/1112 (3.1%)	0.042
Sepsis, n/N(%)	136/1460 (9.3%)	13/133 (9.8%)	123/1327 (9.3%)	0.848
Early Sepsis, n/N(%)	35/1490 (2.3%)	2/140 (1.4%)	33/1350 (2.4%)	0.766
Early Death, n/N(%)	45/1489 (3.0%)	12/140 (8.6%)	33/1349 (2.4%)	0.0006
RDS, n/N(%)	990/1487 (66.6%)	106/139 (76.3%)	884/1348 (65.6%)	0.011
Apgar score <7 at 5 min, n/N(%)	53/1473 (3.6%)	6/136 (4.4%)	47/1337 (3.5%)	0.625

[a] ACS group comparing with Non-ACS group.
[b] Incidence of Brain Injury was calculated among infants with neuroimaging results.
[c]Incidence of Severe ROP was calculated among infants with eye examinations in the NICU.

Abbreviations: ACS, antenatal corticosteroids; BPD, bronchopulmonary dysplasia; IVH, intraventricular hemorrhage; NEC, necrotizing enterocolitis; PVL, periventricular leucomalacia; RDS, respiratory distress syndrome; ROP, retinopathy of prematurity.
