# Supplementary material for: Association of Antenatal Corticosteroids with Neonatal Outcomes among Very Preterm Infants Born to Mothers with Clinical Chorioamnionitis: A Multicenter Cohort Study
Source: Children (Basel). 2024 Jun 3;11(6):680. doi: 10.3390/children11060680 (PMC11202040; doi:10.3390/children11060680)
Supplement: Supplementary file 1 [file children-11-00680-s001.zip › Table S4.pdf]

Table S4. Univariable analysis for association between outcomes and different courses of antenatal corticosteroids among infants born to mothers with clinical chorioamnionitis

**Outcomes**	**Total (N=2166)**	**No ACS (N=227)**	**Single partial course (N=548)**	**Single complete course (N=1111)**	**Repeated courses (N=280)**	***p*-value[a]**
Mortality, n/N(%)	103/2166 (4.8%)	25/227 (11.0%)	22/548 (4.0%)	44/1111 (4.0%)	12/280 (4.3%)	<0.0001
NEC≥Stage II, n/N(%)	81/2166 (3.7%)	7/227 (3.1%)	30/548 (5.5%)	30/1111 (2.7%)	14/280 (5.0%)	0.024
BPD, n/N(%)	569/2160 (26.3%)	79/226 (35.0%)	160/548 (29.2%)	233/1106 (21.1%)	97/280 (34.6%)	<0.0001
Brain Injury, n/N(%)[b]	200/2055 (9.7%)	29/203 (14.3%)	59/529 (11.2%)	80/1061 (7.5%)	32/262 (12.2%)	0.003
Severe IVH, n/N(%)	121/2055 (5.9%)	17/203 (8.3%)	40/529 (7.6%)	45/1061 (4.2%)	19/262 (7.3%)	0.012
cPVL, n/N(%)	102/2055 (5.0%)	17/203 (8.4%)	24/529 (4.5%)	42/1061 (4.0%)	19/262 (7.3%)	0.015
Severe ROP, n/N(%)[c]	54/1802 (3.0%)	12/175 (6.9%)	12/449 (2.7%)	27/934 (2.9%)	3/244 (1.2%)	0.008
Sepsis, n/N(%)	201/2113 (9.5%)	21/216 (9.7%)	61/536 (11.4%)	82/1088 (7.5%)	37/273 (13.6%)	0.006
Early Sepsis, n/N(%)	52/2166 (2.4%)	5/227 (2.2%)	12/548 (2.2%)	27/1111 (2.4%)	8/280 (2.9%)	0.941
Early Death, n/N(%)	66/2160 (3.1%)	16/226 (7.1%)	16/548 (2.9%)	25/1106 (2.3%)	9/280 (3.2%)	0.002
RDS, n/N(%)	1420/2161 (65.7%)	162/225 (72.0%)	346/547 (63.3%)	712/1109 (64.2%)	200/280 (71.4%)	0.014

[a] Overall comparison between 3 groups.
[b] Incidence of Brain Injury was calculated among infants with neuroimaging results.
[c] Incidence of Severe ROP was calculated among infants with eye examinations in the NICU.

Abbreviations: ACS, antenatal corticosteroids; BPD, bronchopulmonary dysplasia; IVH, intraventricular hemorrhage; NEC, necrotizing enterocolitis; PVL, periventricular leucomalacia; RDS, respiratory distress syndrome; ROP, retinopathy of prematurity.
